# Probing cortical excitability using rapid frequency tagging

**DOI:** 10.1016/j.neuroimage.2019.03.056

**Published:** 2019-07-15

**Authors:** A. Zhigalov, J.D. Herring, J. Herpers, T.O. Bergmann, O. Jensen

**Affiliations:** a Centre for Human Brain Health, School of Psychology, University of Birmingham, UK; b Donders Institute, Radboud University Nijmegen, Nijmegen, the Netherlands; c Laboratory for Neurophysiology and Psychophysiology, KU Leuven, Leuven, Belgium; d Department of Neurology and Stroke, and Hertie Institute for Clinical Brain Research, University of Tübingen, Tübingen, Germany; e Institute of Medical Psychology and Behavioral Neurobiology, University of Tübingen, Tübingen, Germany; f Deutsches Resilienz Zentrum (DRZ), Johannes Gutenberg University Medical Center, Mainz, Germany

## Abstract

Frequency tagging has been widely used to study the role of visual selective attention. Presenting a visual stimulus flickering at a specific frequency generates so-called steady-state visually evoked responses. However, frequency tagging is mostly done at lower frequencies (<30 Hz). This produces a visible flicker, potentially interfering with both perception and neuronal oscillations in the theta, alpha and beta band. To overcome these problems, we used a newly developed projector with a 1440 Hz refresh rate allowing for frequency tagging at higher frequencies. We asked participants to perform a cued spatial attention task in which imperative pictorial stimuli were presented at 63 Hz or 78 Hz while measuring whole-head magnetoencephalography (MEG). We found posterior sensors to show a strong response at the tagged frequency. Importantly, this response was enhanced by spatial attention. Furthermore, we reproduced the typical modulations of alpha band oscillations, i.e., decrease in the alpha power contralateral to the attentional cue. The decrease in alpha power and increase in frequency tagged signal with attention correlated over subjects. We hereby provide proof-of-principle for the use of high-frequency tagging to study sensory processing and neuronal excitability associated with attention.

## 1 Introduction

Frequency tagging has been successfully used to study selective stimulus processing in EEG studies (e.g. (Müller et al., 2006, 2003; 1998; Norcia et al., 2015; Vialatte et al., 2010),). The technique has also been applied in MEG studies to investigate visual perception (Parkkonen et al., 2008) as well as the engagement of representational selective areas in the ventral stream (Baldauf and Desimone, 2014). With frequency tagging, a stimulus (usually visual or auditory) is presented at a fixed frequency, which then produces robust steady-state visually evoked potentials or fields (respectively SSVEPs or SSVEFs for EEG and MEG), resulting in a power increase at the tagged frequency (Vialatte et al., 2010). These responses are for instance enhanced by attention (Morgan et al., 1996; Müller et al., 2006) and reflect subjective perception in a bi-stable perception task (Parkkonen et al., 2008). As such they are a useful tool for investigating mechanisms of attention and perception in humans. Typically, frequency tagging is applied at lower frequencies (<30 Hz), which is associated with flicker perception and may interfere with task performance. It also creates a problem when relating frequency tagging to neuronal oscillations in e.g. the alpha (8–13 Hz) and beta band (15–30 Hz) since frequency tagging is likely to entrain or interfere with spontaneous neuronal oscillations as well (Keitel et al., 2014; Spaak et al., 2014). In this study, we use a newly developed projector that allows us to perform frequency tagging at higher frequencies and hence to investigate neuronal excitability and visual attention in relation to endogenous oscillations in the alpha band.

Neuronal oscillations have been shown to play a key role in the processing of sensory information by synchronizing neuronal firing and modulating synaptic input (Schroeder and Lakatos, 2009). For example, alpha oscillations have been hypothesized to support active inhibition of brain regions processing task-irrelevant, and possibly distracting, stimuli (Foxe and Snyder, 2011; Jensen and Mazaheri, 2010; Klimesch et al., 2007). This is underscored by the findings that posterior alpha oscillations are strongly modulated by spatial attention (Händel et al., 2011; Thut et al., 2006; Worden et al., 2000). Additionally, the phase of alpha has been shown to modulate perception (Mathewson et al., 2011; Vanrullen et al., 2011) and cortical excitability (Dugué et al., 2011; Scheeringa et al., 2011; Spaak et al., 2012).

In this study, we apply frequency tagging between 60 and 80 Hz in order to probe neocortical excitability in relation to alpha oscillations. A previous study by Christoph Hermann (Herrmann, 2001) has shown that rapidly flickering LED can drive the visual cortex as measured by human EEG up to around 100 Hz. Intracranial recordings in monkeys and humans have demonstrated that neuronal spiking in visual regions is entrained by the refresh rate of a CRT computer monitor (60 Hz) (Krolak-Salmon et al., 2003; Sandström et al., 1997; Williams et al., 2004). We applied frequency tagging above 60 Hz using a projector with a 1440 Hz refresh rate while recording whole-head MEG. This was done while subjects attended to flickering face and house stimuli in a cued spatial attention paradigm. The aim was to determine if cortical excitability as modulated by spatial attention could be estimated using rapid frequency tagging. Our core assumption is that the amplitude of MEG signal at the tagged frequency reflects neuronal excitability. Furthermore, we expect neuronal excitability to increase with spatial attention and thus the tagged signal as well. A second aim was to investigate the relationship between alpha band oscillations and the cortical excitability assessed by rapid frequency tagging.

## 2 Materials and Methods

### 2.1 Participants

Participants were recruited from a participant database of the Radboud University Nijmegen. Twenty-five healthy (17 females, aged 26 ± 10 (mean ± SD)) participants partook in the study. Two of the subjects were excluded due to an excessive amount of rejected trials. Written informed-consent was acquired before enrolment in the study. All subjects conformed to standard inclusion criteria for MEG experiments. Subjects had normal or corrected-to-normal vision. The study was approved by the local ethics committee (CMO region Arnhem/Nijmegen). Subjects received financial compensation of 8 euros per hour or were compensated in course credits.

### 2.2 Attention task

**Fig. 1:**
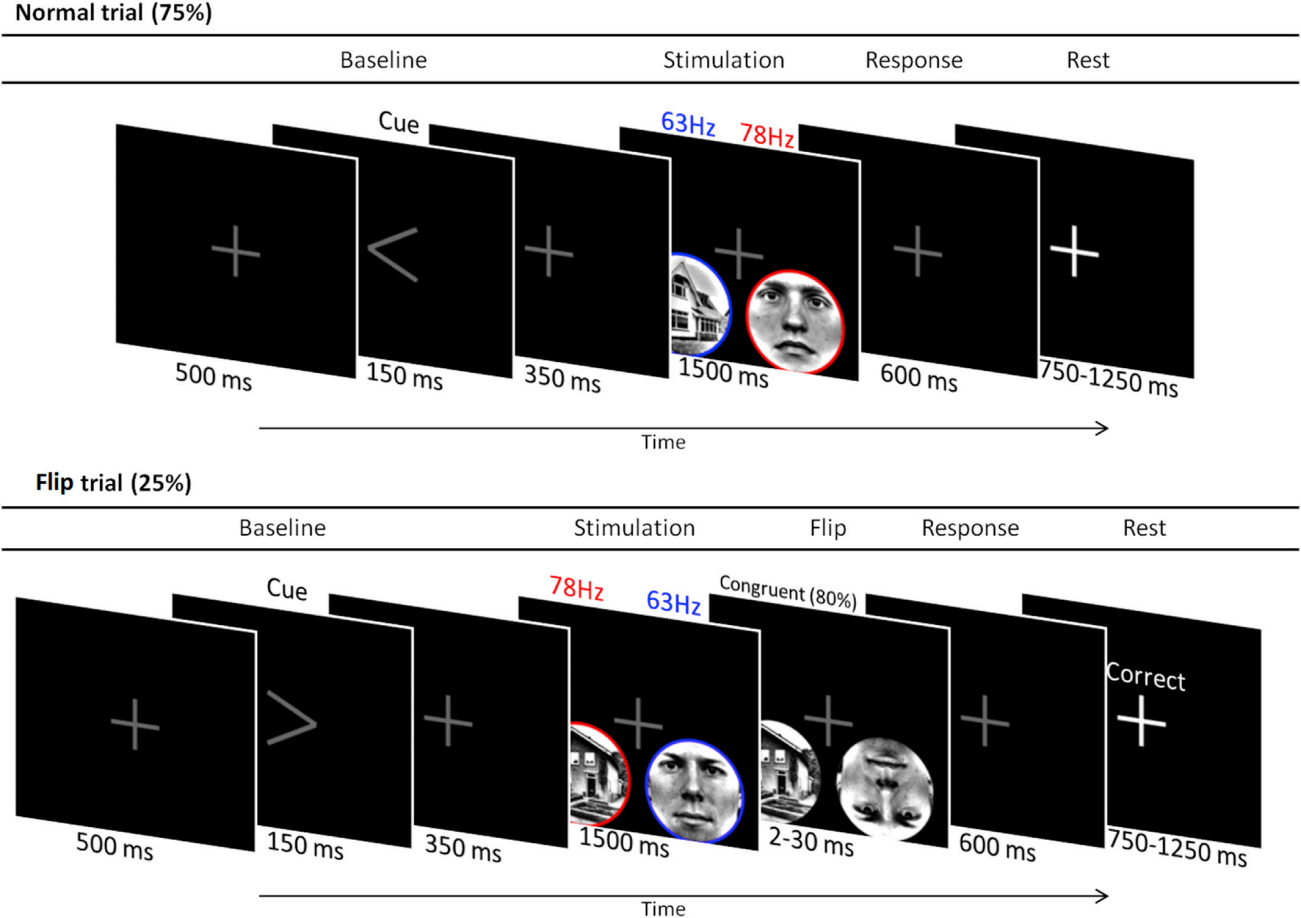
Schematic representation of the experimental paradigm. After an attentional cue, a house-face pair was presented at 63 and 78 Hz (counterbalanced over trials). In 20% of the trials, one of the images was flipped vertically and required participant's response. In 5% of the trials (catch trials), the flip was in the hemifield opposite to the cued side and participants had to ignore this event.

Participants performed a spatial attention task (4 blocks of 15 min) in which they had to allocate attention to either the left, or the right visual hemifield, depending on a cue presented at the start of each trial (Fig. 1).

Each trial started with a fixation cross (500 ms) followed by an arrow (150 ms) indicating the hemifield that the participants had to attend to (attentional cue), while fixating on the center of the screen. The fixation cross was shown for 350 ms after the attentional cue, and then stimuli were presented in the left and right visual hemifield for 1500 ms. Participants were instructed to detect a vertical flip of the attended stimulus. Flips occurred at the end of trial in 25% of trials. In 20% of these trials, the flip was on the cued side, while in 5% of the trials (catch trials), the flip was in the hemifield opposite to the cued side, and participants had not to respond. Participants responded to the vertical flip by button presses with either index finger (flip on the left) or middle finger (flip of the right). The duration of the flipped stimulus was adjusted using QUEST adaptive staircase procedure (Watson and Pelli, 1983) to attain 80% correct responses. The initial duration of the flipped stimuli was 10 ms and it varied between 2 and 30 ms during the session controlled by the QUEST procedure. The validity of the responses was indicated on the screen as correct (“CORRECT”), incorrect (“INCORRECT”), or missed (“MISS”) response. Next trial began following a random interval of 500 ± 250 ms. Such relatively short inter-stimulus interval may influence the neuronal responses in the subsequent trials; however, the random stimulus onset reduces this effect. The experimental paradigm was implemented in MATLAB 2017b (Mathworks Inc., Natrick, USA) using Psychophysics Toolbox 3.0.11 (Kleiner et al., 2007).

### 2.3 Visual stimuli

Pairs of stimuli (face and house) were presented simultaneously in the lower left and right visual field (8.3° eccentricity). Different combinations of faces and houses (comprising ten faces and ten houses) were presented in random order over the trials. Luminance of the grayscale stimuli was normalized using the SHINE Toolbox for MATLAB (Willenbockel et al., 2010) and a circular mask was applied to the images (see, Fig. 1). Stimuli were presented at a rate of respectively 63 Hz and 78 Hz (counter-balanced over trials). The presentation rate was achieved by modulating transparency of the stimulus with a sinusoid at the target frequency, phase-locked across trials. Direction of attention, pairing of face-house stimuli and tagging frequencies were counterbalanced over trials.

### 2.4 Projector

To achieve a high rate of stimuli presentation, we used a GeForce GTX960 2 GB graphics card in combination with a PROPixx DLP LED projector (VPixx Technologies Inc., Saint-Bruno-de-Montarville, Canada). This projector provides a refresh rate up to 1440 Hz by dividing each frame received from the graphics card (at 120 Hz) into multiple frames. Basically, the projector divides each received frame (1920 x 1200 pixels) into four equally sized quadrants (960 x 600 pixels), allowing for a fourfold increase in refresh rate (480 Hz). Colour (RGB) images presented in each quadrant can be further converted to a grayscale representation by equalizing all components of RGB code. As such, this allows for an increased refresh rate of 120 Hz by a factor of 4 times 3 (1440 Hz) when presenting grayscale images with a resolution of 960 x 600 pixels.

### 2.5 MEG data acquisition

MEG was acquired using a 275-sensor axial gradiometer CTF system (CTF MEG systems, Coquitlam, Canada). The MEG data was low-pass filtered at 300 Hz using embedded anti-aliasing filters and sampled at 1200 Hz. Head position of the participants was continuously monitored throughout the experiment using three head-localization coils placed on the nasion and both periauricular points (Stolk et al., 2013).

### 2.6 MEG data preprocessing

MEG data were analysed using MATLAB and the Fieldtrip toolbox (Oostenveld et al., 2011). The data were segmented into 3.5 s epochs; −1.5–2 s relative to the onset of flickering stimulation. The data were further down-sampled to 300 Hz and ICA unmixing matrices were calculated using the ‘infomax’ algorithm (Makeig et al., 1996) on the first 90 principal components of the data. Components containing topographies and time courses clearly matching cardiobalistic activity and eye-blinks were rejected from the data. The trials containing large amplitude events (above 5 SD) were rejected. The number of such trials has not exceeded of 5% of total amount of trials.

### 2.7 Sensor-level analysis

Synthetic planar gradients were calculated to ease interpretation of the topography of power measurements (Bastiaansen and Knösche, 2000). The planar gradient power was combined by summing the orthogonal components for each sensor location.

To estimate the effect of attention on power at the tagging frequencies or neuronal oscillations, the attention modulation index (AMI) was calculated. To this end, spectral power for time-frequency representations (TFR) was computed using Fourier transform (FT) for each sensor and epoch from −1.5–2 s relatively to stimulus onset. The spectral power was computed for multiple moving-time windows (1 s length and 0.05 s step) weighted by the Hanning taper, and over a range of frequencies (1–100 Hz). The effect of spatial attention for the left sensors was calculated as follows:

(1) AMI_{SL}_ = (P_AR{SL}_ – P_AL{SL}_) / (P_AR{SL}_ + P_AL{SL}_)

where SL denotes the subset of left sensors (similarly, SR denotes the subset of right sensors); P_AL_ and P_AR_ denote spectral power averaged over trials “attention left” and “attention right”, respectively. The AMI for the right sensors was computed in the same manner, and the resulting AMI was obtained by combining AMI for the left and right sensors with inverse polarity as follows:

(2) AMI = AMI_{SL}_ – AMI_{SR}_

In case of all sensors AMI (see, Fig. 4), we computed the spatial patterns as follows:

(3) AMI = (P_AR_ – P_AL_) / (P_AR_ + P_AL_)

where P_AL_ and P_AR_ denote spectral power averaged over trials “attention left” and “attention right”, respectively.

### 2.8 Statistical comparisons

Unless specified otherwise, conditions were compared using two-sided paired-sample t-tests. To statistically quantify the AMI in spatial domain, we used cluster-based permutation statistics (Maris and Oostenveld, 2007), which allow controlling for multiple comparisons over sensors.

## 3 Results

Subjects performed a cued spatial attention task and were instructed to press a button if a stimulus flipped vertically on the cued side (left or right). In each trial, a pair of face/house stimuli appeared for 1.5 s in the left and right visual hemifield (Fig. 1). Each stimulus was flickering at either 63 Hz or 78 Hz. The location of the face and house stimulus (left or right hemifield), tagging frequency (63 or 78 Hz), and direction of attention were counterbalanced over trials throughout the experiment.

### 3.1 Behaviour

Behavioural results demonstrated that participants were able to detect flips in the attended hemifield while ignoring flips in the unattended hemifield. The average hit rate was 0.75 ± 0.05 (mean ± SD) and the average reaction time was 0.47 ± 0.03 s (mean ± SD).

### 3.2 Spatial attention modulates responses of frequency-tagged stimuli

**Fig. 2:**
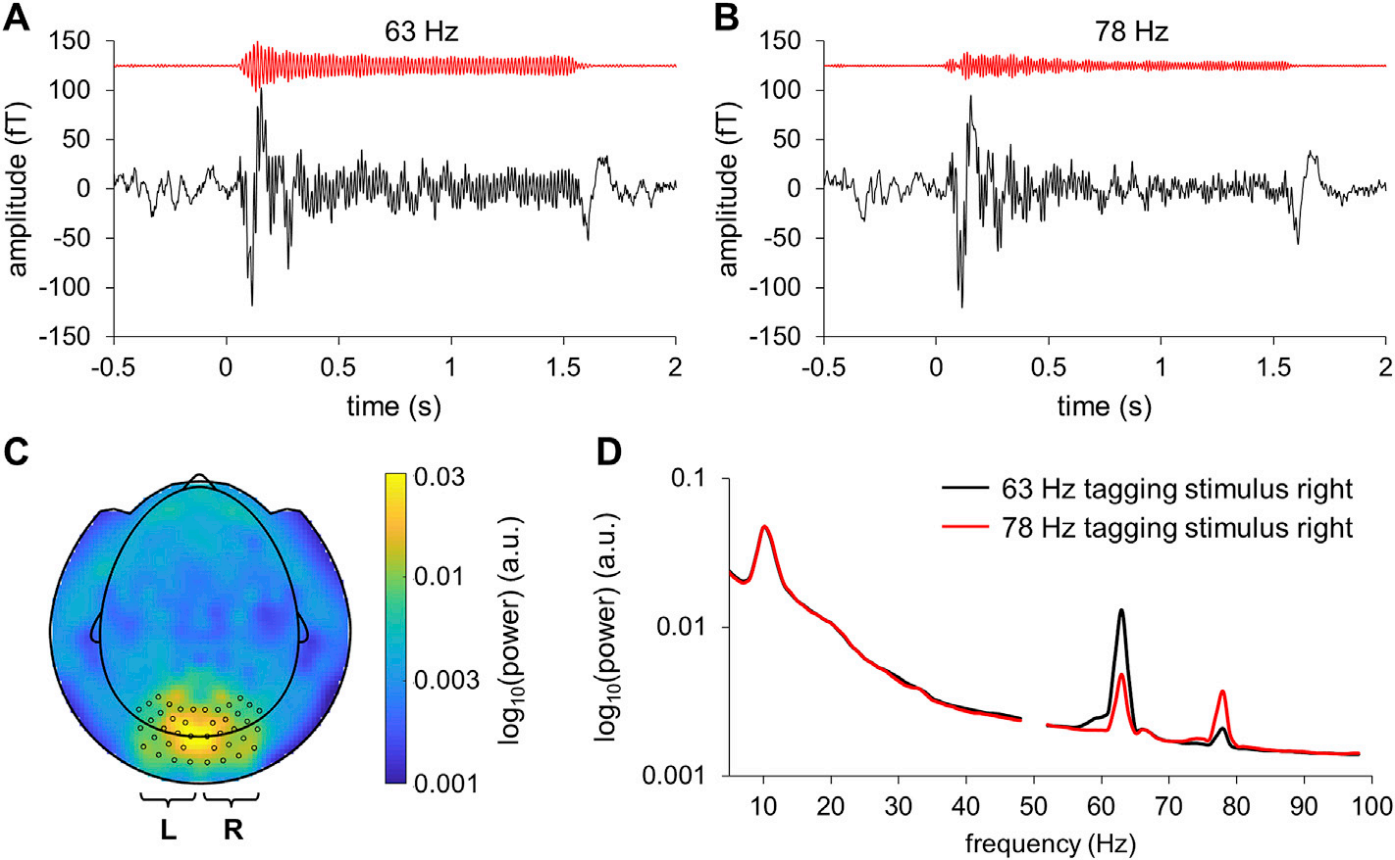
Event-related fields for a representative participant showed clear responses at the tagging frequencies. Note that the frequency tagged signals were presented with the same phase over trials. (A) Broadband (black line) and narrowband (red line) trial-averaged ERFs for 63 Hz stimulus (presented right) for the left occipital sensors (see panel C). (B) Trial-averaged ERFs for 78 Hz stimulus (presented right) for the left occipital sensors. (C) Left and right occipital MEG sensors that covered areas with the stronger power at the tagging frequencies for all the participants were used in the analysis. (D) Normalized group-level power spectra for the left sensors when the tagged image was presented at 63 Hz and 78 Hz in the right hemifield. Prior to computing individual power spectra, the trials were normalized by the standard deviation of time series over sensors. The line noise with peak near 50 Hz was cut out in the plot.

To assess the response in the early visual cortex to the flickering stimuli, we calculated time-locked averages of the event-related fields pooling data over stimulus type (face, house) and direction of attention (left, right). Visual stimulation at the tagging frequencies produced clear steady-state visual evoked fields (SSVEFs) in occipital sensors (Fig. 2A and B). The SSVEFs lasted for the entire stimulation period and were markedly larger for 63 Hz compared to 78 Hz, as evident by a significant main effect of tagging frequency.

We calculated the power spectra for each trial and then averaged over the trials. The sensors were selected according to the strongest response at the tagging frequencies for all the participants (Fig. 2C). The group-level normalized power spectra showed pronounced peaks in the tagging frequencies at the selected occipital sensors (Fig. 2D), suggesting that the frequency tagging method produces reliable responses in majority of the participants.

**Fig. 3:**
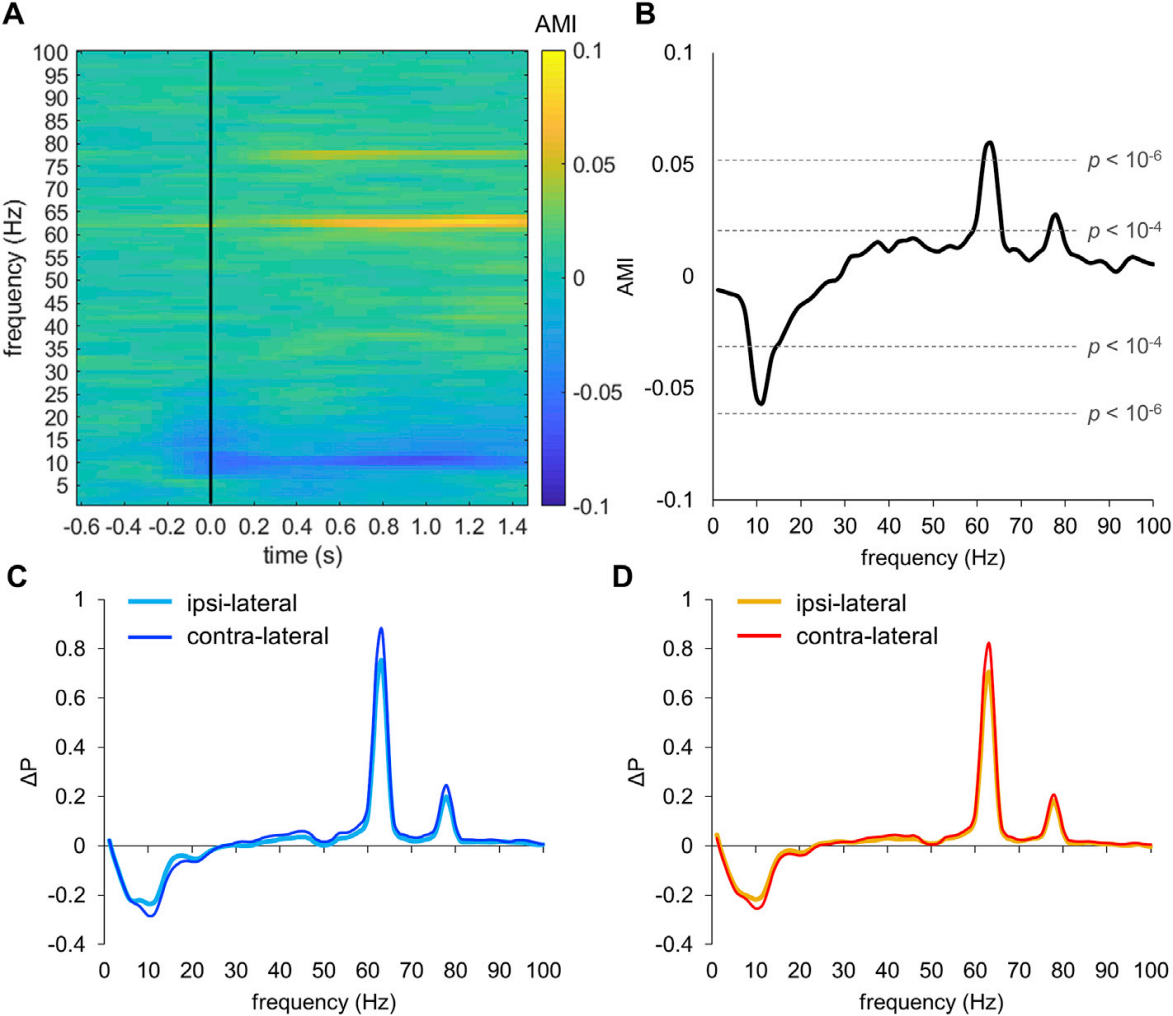
Attention modulates power in the alpha band and at the tagging frequencies. (A) Time-frequency representation of the attention modulation index (AMI). The AMI reflects the power modulation in the sensors contra-versus ipsilateral to the attended hemifield for combined left and right occipital sensors (see Fig. 2C for sensors selection). The power was calculated per trial and then averaged. Black line indicates onset of the frequency tagged stimuli; the cue onset was at −0.5 s. (B) The AMI (averaged over time bins 0.5–1.5 s) at the group level. Dashed lines indicate *p*-values of the *t*-test comparing modulation index against zero (over participants). The effect is highly robust in the 8–12 Hz alpha band and at 63 and 78 Hz even if multiple comparisons over frequencies are considered. (C) Relative power change compared to the baseline (−1, −0.5 s) at the left sensors for trials “attention left” (cyan line; ipsilateral to the cue) and “attention right” (blue line; contralateral to the cue). (D) The same as (C) but for the right sensors for trials “attention right” (orange line; ipsilateral to the cue) and “attention left” (red line; contralateral to the cue).

To quantify the effect of attentional modulation of power at the tagging signals we calculated the attention modulation index (AMI; see Materials and Methods). The AMI indicates the power at sensors contralaterally to the attended hemifield minus the power ipsilaterally (normalized by the sum); as such the figures reflect attention ‘*ON*’ minus attention ‘*OFF*’. The AMI was computed for the entire trial interval from −1.5–2 s (relatively to stimulus onset) using time-frequency representations of power (Fig. 3A). This was done for the sensors shown in Fig. 2C. The signals at the tagged frequencies increased with attention; i.e. they increased in the hemisphere contralateral to the attended hemifield. The alpha power was relatively suppressed in the hemisphere contralateral to attention. The AMI was then averaged over time bins in the 0.5–1.5 s interval to reduce the contribution of the initial evoked response (Fig. 3B). The AMI was significantly different from zero (t_22_ > 5.64, *p* < 10^−5^, uncorrected) in both the alpha band and at the tagging frequencies. However, AMI at 63 Hz was significantly larger than AMI at 78 Hz (t_22_ = 2.74, *p* < 0.01), suggesting that the efficacy of the response decreases at high frequencies (above 20 Hz) as a function of (tagging) frequency.

The AMI was derived as a difference in power between trials “attention left” and “attention right” (see, equation (1)), and hence, it does not indicate whether the difference is related to ipsilater increase or contralateral decrease in power at the alpha frequency (and opposite in the tagging frequencies). To clarify this, we quantified the relative change in power compared to the baseline as follows, ΔP = (P_stimulation_ – P_baseline_)/P_baseline_, where P_baseline_ and P_stimulation_ denote power at the baseline and stimulation, respectively. The power at the alpha frequencies showed larger decrease contralaterally to stimulation side and the power at the tagging frequencies showed an opposite change (Fig. 3C and D).

**Fig. 4:**
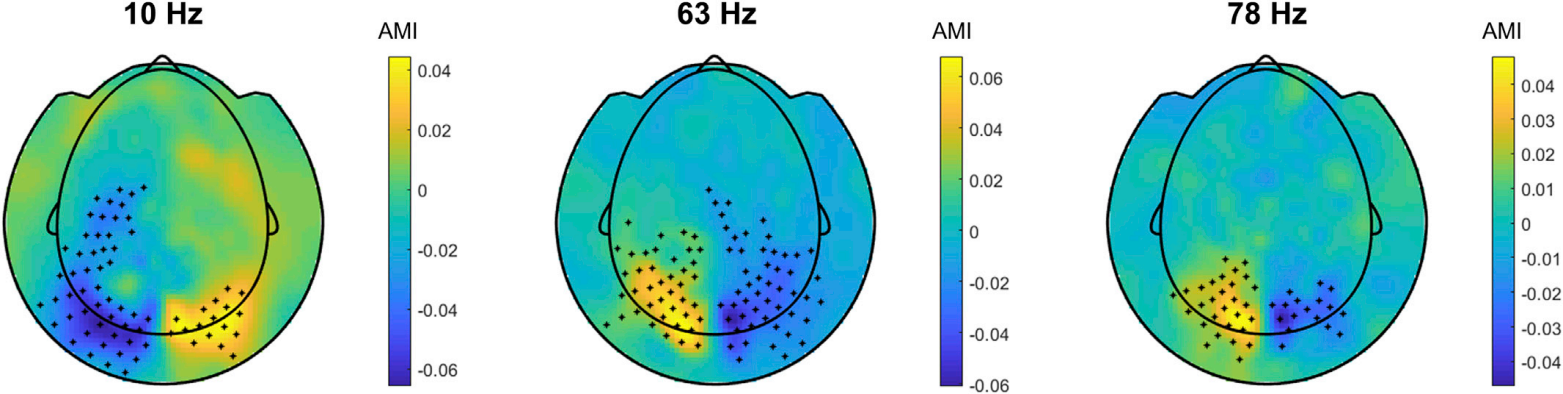
Group average topography maps of the AMI in the alpha band (10 ± 2 Hz) and tagging frequencies (63 and 78 Hz). Black dots indicate MEG sensors at which amplitude modulation index was significantly different from zero (*p* < 0.05, cluster-based permutation).

Using cluster-based permutation test controlling for multiple comparisons over sensors (see Materials and Methods), we identified the clusters of sensors at which power was significantly modulated by attention (Fig. 4). The spatial clusters of AMI in the alpha band and the tagging frequencies were over occipito-parietal areas; however, the alpha frequency clusters were located more posterior compared to those of the tagging frequencies. We quantified the overlap between clusters using the Jaccard (or Intersection over Union) index. The results of such method should be taken with caution because the cluster size is strongly affected by the signal-to-noise ratio and by the metric of statistical testing. The spatial clusters at the alpha and higher tagging frequency showed a moderate (nearly 60%) overlap as indicated by the Jaccard index. The spatial map of AMI at the alpha frequency was well in line with previous observations (e.g. (Foxe and Snyder, 2011; Händel et al., 2011; Thut et al., 2006; van Ede et al., 2011; Worden et al., 2000),), suggesting that the spatial attention related modulations of alpha activity are preserved despite the frequency tagging. Clusters at the lower (63 Hz) and higher (78 Hz) tagging frequencies showed a strong (over 90%) overlap as indicated by the Jaccard index; however, the clusters at 63 Hz were slightly larger compared to those for the higher frequency (78 Hz).

### 3.3 Relationship between AMI at the alpha and tagging frequencies

**Fig. 5:**
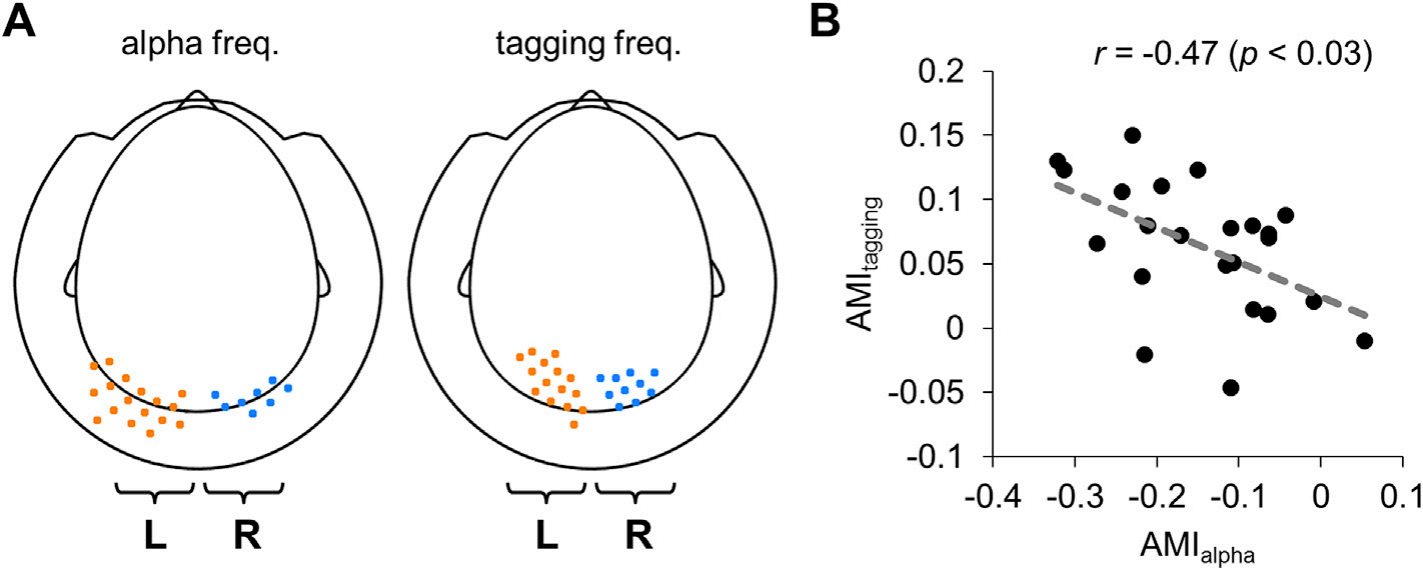
Relationship between the modulation of alpha power and frequency tagging. (A) Spatial masks for the alpha and tagging frequencies. The masks were obtained by selecting sensors expression the 10% of largest absolute AMI values. (B) Scatter plot of individual AMI relating the alpha power modulation and the power combined for the tagging frequencies. Subjects with a strong alpha power modulation with attention were also subjects with a strong modulation of the tagged signals.

Considering the inverse relationship between the attentional modulation in the alpha band and the tagging frequencies (see Fig. 4), we tested whether participants with a stronger modulation of alpha power have stronger power modulation at the tagging frequencies. To this end, we derived the individual AMI of the alpha band and the tagging frequencies (63 and 78 Hz combined) and assessed their correlation over subjects. We defined separate masks for the alpha and tagging frequencies (Fig. 5A) by selecting sensors expressing the 10% of largest absolute AMI values (see Fig. 4). We observed a robust correlation (*r* = −0.47, *p* < 0.03; Spearman correlation) between individual AMIs (Fig. 5B). This suggests that participants demonstrating stronger alpha modulation had also stronger modulation at the tagging frequencies. Additionally, we assessed the Spearman correlation between the individual AMI of the alpha and each tagging frequency, separately. The correlation was significant for the lower tagging frequency (63 Hz; *r* = −0.51, *p* < 0.01), but it was not significant for the higher tagging frequency (78 Hz; *r* = −0.24, *p* > 0.26). This result could be partially explained by lower signal-to-noise ratio at the higher frequencies.

To test whether the relationship between AMI at the alpha and tagging frequencies holds at the single trial level, we computed the lateralization index (LI) for each trial as follows,

(4) LI_(*i*)_ = (P_(*i*){SR}_ – P_(*i*){SL}_) / (P_(*i*){SR}_ + P_(*i*){SL}_),

where P_(*i*)_ denotes power for trial (*i*), SL and SR denote indices of the left and right sensors, respectively. In contrast to equation (1), we subtracted left and right sensors instead of “attention left” and “attention right” trials. The LI_(*i*)_ were split into two categories “attention left” and “attention right”, and correlation (and median split *t*-test) between LI at the alpha and tagging frequencies was computed for each category separately. We did not find any significant (*p* > 0.05) correlation (or median split t-statistics) between LI at the alpha and tagging frequencies. A larger amount of trials is necessary to establish whether such a relationship exists or not.

## 4 Discussion

We here demonstrate that tagging of visual stimuli at rapid frequencies (63 and 78 Hz) can induce neuronal responses at the same frequencies in visual cortex. Spatial attention towards a visual object produced stronger responses at the tagging frequency contralateral to the direction of attention compared to the unattended stimulus. As such, the tagging signal reflects the gain of neuronal excitability with spatial attention. Posterior alpha oscillations decreased in magnitude in posterior regions contralateral compared to ipsilateral to the direction of attention. This demonstrates that the alpha oscillations were not disrupted by the tagging signal.

The correlation between individual modulations in the alpha and the power at the tagging frequencies suggests a link between attentional mechanisms for the alpha power and tagging frequencies. One possibility is that alpha modulated by attention determines the neuronal excitability which then determines the increase in the frequency tagged responses. This interpretation however only partially explains the correlation as the topographies of AMI at the alpha and tagging frequencies did not perfectly overlap.

### 4.1 Proof-of-principle: using rapid frequency-tagging to probe neocortical excitability

This study provides proof-of-principle that rapid frequency tagging can be used to probe brain mechanisms involved in processing of visual stimuli without affecting endogenous oscillations in the alpha range. Previous studies have shown that it is possible to elicit responses in early visual cortex by using flickering light emitting diodes (LED) at frequencies up to 100 Hz (Herrmann, 2001). However, the use of discrete LEDs does not allow for creating complex stimuli. In this study, we used a state-of-the-art LED projector that is capable of presenting stimuli at a refresh rate of 1440 Hz. Thus, this projector allowed us to modulate luminance of the stimulus at frequencies up to 720 Hz (the Nyquist frequency of the projector). Similarly to the study of Herrmann (2001), we observed weaker neuronal response for the stimuli tagged at 78 Hz compared to 63 Hz, although both stimuli were modulated with the same intensity. This might be explained by the attenuation resulting from the synaptic drives in the early visual stream. The time course of the post-synaptic potentials are in the order of ∼10 ms (Koch et al., 1996), which effectively creates a ∼100 Hz low pass filter. Another possibility is that the proximity of the frequency of the tagged signal to the frequency of the individual gamma oscillations influences the magnitude of the tagged response. These possibilities require further investigation in future studies where the tagging over a broader frequency is systematically explored.

### 4.2 Attention enhances neural response to tagging signal

An assumption underlying the use of frequency tagging as a tool to study sensory processing in the brain is that the EEG/MEG signal at the tagged frequency reflects underlying sensory processing. We have shown here that spatial attention modulates power at the tagging frequency in the expected direction; the response at the tagged frequency was enhanced when attention was directed towards the stimulus and suppressed when attention was directed away. This suggests that the gain increase associated with the allocation of spatial attention results in increased neuronal excitation, which in turn is reflected by the power of the frequency tagged MEG signal.

### 4.3 Alpha oscillations are not disrupted by rapid frequency tagging

The increase in neuronal response modulated by spatial attention has also been shown at the lower (up to 30 Hz) tagging frequencies (e.g. (Müller et al., 2006; Toffanin et al., 2009)). However, frequency tagging at lower frequencies (0.5–30 Hz) is likely to interfere with endogenous neuronal oscillations. Most frequency tagging experiments are limited to frequency bands below 30 Hz (e.g. (Müller et al., 2006; Norcia et al., 2015; Toffanin et al., 2009)). In this case, the tagging signal produces visible a flicker and may potentially entrain the ongoing oscillations (Spaak et al., 2014; Thut et al., 2011). This is especially evident given that tagging produces the strongest neuronal response in the visual system at frequencies between 12 Hz and 18 Hz (Kuś et al., 2013).

In our study, alpha oscillations in the posterior regions remained undisrupted by the rapid frequency tagging. Alpha power increased ipsilaterally to the direction of attention and decreased contralaterally as observed in numerous other studies (Händel et al., 2011; Thut et al., 2006; Worden et al., 2000). Applying frequency tagging at higher frequencies therefore makes is possible to in conjunction study the role of lower-frequency oscillations on sensory processing.

In future work it would be interesting to investigate if the rapid frequency tagging entrains intrinsic gamma oscillations or rather reflect a simple feedforward drive. Similar considerations have been put forward for the alpha rhythm (Keitel et al., 2014). It would also be interesting to investigate the relationship between the phase of the alpha oscillations and the frequency tagged signal. Indeed the phase of alpha oscillations has been suggested to modulate perception rhythmically in a pulsed inhibitory manner; and this modulation is dependent on attention (Kizuk and Mathewson, 2017). This notion could be investigated in the context of a phase-code coordinated by the alpha rhythm as proposed by Jensen and colleagues (Jensen et al., 2014).

### 4.4 Does rapid frequency tagging entrain neuronal gamma oscillations?

There are several studies (Adjamian et al., 2004; Murty et al., 2018; Muthukumaraswamy and Singh, 2013) that attempted to apply stimulation at frequencies in the gamma range in order to entrain endogenous gamma band oscillations (30–90 Hz). Such studies are important for understanding the important function gamma band oscillations may have in neuronal computations (Fries et al., 2007; Jensen et al., 2007; Varela et al., 2001). Bauer and colleagues (Bauer et al., 2009) showed that attention could be captured by subliminally perceived stimuli flickering at 50 Hz. Manipulating visual perceptual integration by modulating the phase of externally driven gamma frequency stimulation has proven difficult (Bauer et al., 2012). Future studies may explore to what extent the neuronal activity elicited by rapid frequency tagging entrains endogenous gamma oscillations. If this is the case, frequency tagging should be more efficient and result in a relative power increase when applied at the frequency of the individual endogenous gamma oscillations. This could also be investigated by pharmacological means. It is well established that GABAergic inhibition from interneurons plays a crucial role for generating of gamma oscillations (Traub et al., 1999). In support of this notion, we recently demonstrated that visual gamma oscillations in humans increase when the GABergic agonist Lorazepam is applied (Lozano-Soldevilla et al., 2014). If rapid frequency-tagging entrains natural gamma oscillations, one would expect that rapid-frequency tagging in the gamma band increases with the application of GABAergic agonists.

## 5 Conclusion

We set out to investigate the feasibility of rapid frequency tagging to study the role of sensory processing in the visual cortex. Our results show that it is indeed possible to measure responses at the tagging frequencies and that these responses are modulated by spatial attention. The modulation of alpha power was inversely related to the modulation in gamma power. These findings provide important proof-of-principle that rapid frequency tagging can be used to measure neuronal excitability of visual cortex in a stimulus specific manner to for instance investigate spatial attention. Furthermore, the dynamical properties of the alpha band oscillations were preserved despite the frequency tagging. Rapid frequency tagging is highly advantageous to conventional frequency tagging at lower frequency (<20 Hz) as it does not produce a visible flicker and furthermore the faster frequencies allow for investigating the tagged response with a better temporal resolution. The stage is now set for applying frequency tagging in combination with EEG or MEG to study the dynamical properties of the visual system.
